# Timeliness of Childhood Primary Immunization and Risk Factors Related with Delays: Evidence from the 2014 Zhejiang Provincial Vaccination Coverage Survey

**DOI:** 10.3390/ijerph14091086

**Published:** 2017-09-20

**Authors:** Yu Hu, Qian Li, Yaping Chen

**Affiliations:** Institute of Immunization and Prevention, Zhejiang Center for Disease Control and Prevention, Hangzhou 310000, China; zjmyslq@163.com (Q.L.); zjmyscyp@163.com (Y.C)

**Keywords:** primary immunization, vaccination, timeliness, coverage, risk factors

## Abstract

*Background:* this study aimed to assess both immunization coverage and timeliness, as well as reasons for non-vaccination, and identity the risk factors of delayed immunization, for the vaccines scheduled during the first year of life, in Zhejiang province, east China. *Methods:* A cluster survey among children aged 24–35 months was conducted. Demographic information and socio-economic characteristics of the selected child, the mother, and the household were collected. Immunization data were transcribed from immunization cards. Timeliness was assessed with Kaplan–Meier analysis for each vaccine given before 12 months of age, based on the time frame stipulated by the expanded program on immunization of China. Cox proportional hazard regression was applied to identify risk factors of delayed immunization. *Results:* A total of 2772 eligible children were surveyed. The age-appropriate coverage ranged from 25.4% (95% CI: 23.7–27.0%) for Bacillus Calmette–Guerin (BCG) to 91.3% (95% CI: 90.2–92.3%) for the first dose of oral poliomyelitis vaccine (OPV1). The most frequent reason for non-vaccination was parent’s fear of adverse events of immunization. Delayed immunizations were associated with mother having a lower education level, mother having a job, delivery at home, increasing number of children per household, and having a lower household income. *Conclusions:* Although the timeliness of immunization has improved since 2011, necessary steps are still needed to achieve further improvement. Timeliness of immunization should be considered as another important indicator of expanded program on immunization (EPI) performance. Future interventions on vaccination coverage should take into consideration demographic and socio-economic risk factors identified in this study. The importance of adhering to the recommended schedule should be explained to parents.

## 1. Introduction

To achieve effective control of vaccine preventable diseases (VPDs), a high coverage with efficacious vaccines is required. The goal of the World Health Organization (WHO) for childhood immunization coverage is 90% [1,2]. In addition to high coverage, timeliness of vaccination is also important for the success of the expanded program on immunization (EPI), especially a timely start of immunization for the first year of life, as the maternal antibody declines rapidly [3]. It is necessary to receive vaccines at the recommended ages, which are normally based on the youngest age groups at risk for specific infections, when the profiles of vaccine safety and efficacy have been demonstrated [4,5]. Delayed vaccination increased the risk time between the loss of maternal antibodies and the protection from vaccine-induced immunity, which negatively affects the herd immunity and contributes to the outbreaks of VPDs [6].

Immunization coverage is the most frequently used indicator for immunization program evaluation [7]. Administrative coverage is calculated as dividing the number of vaccine doses administered (numerator) by the number of children in the target age group (denominator). This calculation is usually influenced by data quality issues, as the denominator is often inaccurate [7], and it is impossible to evaluate the timeliness of vaccination, as it does not include information on the exact age of vaccination. Timely vaccination can be assessed through the Kaplan–Meier method and the Cox proportional hazard regression, which are the survival analysis techniques for describing the time-to-event data [8,9,10,11].

Zhejiang province is on the east coast of China. It covers an area of 101,800 km^2^ and is one of the most densely populated provinces in China, with a population of 72 million (2012 census). The annual population growth of Zhejiang province is around 10%, with an estimated 713,261 births in 2012. Administratively, it is divided into 11 cities, 90 counties, and 1319 towns. In Zhejiang province, the EPI schedule of primary immunization includes the following vaccines: Bacillus Calmette–Guerin (BCG) at birth; hepatitis B vaccine (HBV) at birth, one month and six months; oral poliomyelitis vaccine (OPV) at two, three, and four months; diphtheria, tetanus, pertussis combined vaccine (DTP) at three, four, and five months; and measles and rubella combined vaccine (MR) at 8 months [12]. All vaccines included in the EPI schedule are offered freely to the children by the government. Immunization coverage survey is used to monitor the coverage, and it is compared with data from administrative coverage reporting when answering specific questions to guide the program strategies.

In 2011, Zhejiang provincial center for disease control and prevention (CDC) conducted a coverage survey in 18 counties, and 1146 children born from 1 January 2008 to 31 December 2009 were investigated. The results of this study showed that the coverage of the primary vaccination doses could reach the goal of 90% set by the Chinese EPI. However, the coverage of timely vaccination, which meant administration within one month after the due date, ranged from 43.7% to 59.3% [12]. Furthermore, another coverage survey was conducted among 718 migrant children aged 8–48 months in 2011 in Yiwu city, where the proportion of migrant people was over 60%. The results of this survey showed that for MR the coverage was 76.9% but the timely vaccination coverage was only 7.5% [13].

Obviously, there was still scope and a need to increase the timeliness of vaccination to reduce the risk of VPDs infection. However, the determinants on untimely vaccination have scarcely been reported in China, which hinders the development of effective interventions or strategies. As such, a coverage survey was conducted in 2014 among children aged 24–35 months in Zhejiang province to assess the coverage and timeliness, for the vaccines doses scheduled during the first year of life. Furthermore, we also explored the reasons for the non-vaccination and the predictors of delayed or missed immunization.

## 2. Methods

### 2.1. Target Children

Between 29 October and 6 November 2014, a household-based cluster survey was conducted among children aged 24–35 months (born from 1 September 2011 to 31 August 2012) living in Zhejiang province. We chose this age range to ensure that all eligible children had a chance of having completed the primary vaccination scheduled before the end of the first year of life. 

### 2.2. Sample Size

The sampling procedure of this study was based on the immunization cluster survey recommended by WHO [14]. The formula used to estimate the sample size was as followings: $N_{\min}=deff\times \frac{z_{\left(1-\alpha /2\right)}^{2}\times p\times \left(1-p\right)}{d^{2}}$. To reach the estimates of coverage at city level with a two-tailed *α* error of 5% and a permissible error (*d*) of 0.08, assuming the expected timely vaccination coverage (*p*) at 70% and a design effect (*deff*) of 2, the minimum sample size required for each city was 252 eligible children, divided in 6 clusters (towns) of 42 children in each cluster, corresponding to 2772 children in the entire province.

### 2.3. Sampling Procedures in the Field

First, six towns (clusters) of each city were selected from the list of towns (with the population of towns) by city, on the basis of the probability proportional to population size (PPS) [15]. Second, one community was randomly selected through simple ballot from the list of all communities of each selected town. Third, the first household was selected randomly from the list of all households in the selected community, by using the table of random numbers. Fourth, we selected the subsequent 41 households, by turning to the right while exiting the household, and visiting the adjacent one. Only one eligible child per household was randomly selected for the survey. The household was excluded if there were no eligible children or it appeared vacant. Households in which somebody was living, but without any response, were re-scheduled for another visit. If we could not find 42 eligible children in the selected community, then we moved to the closest community in the same town and repeated the procedures above to survey the remaining children.

### 2.4. Data Collection

A pre-tested questionnaire, which was designed to take no more than 15 min, had been developed by Zhejiang provincial CDC. Parents of the selected children were visited at home by the trained interviewers. Immunization data were transcribed from the immunization cards and validated through Zhejiang provincial immunization information system (ZJIIS), the electronic immunization registry established in 2005 in Zhejiang province [16]. Demographic information and socio-economic characteristics of the selected child, the mother, and the household were collected. Furthermore, reasons for the non-vaccination were also collected.

### 2.5. Measurements

We defined non-vaccination as any child without written evidence of having received the specific vaccination from either the immunization card or the vaccination record in ZJIIS.

In this study, we considered three definitions of immunization coverage. First, we defined the general coverage as the proportion of children having received primary vaccination, independent of their age at vaccination. The full immunization (FI) coverage was defined as the proportion of children that received all of the 11 vaccinations. Second, we defined the acceptable timely coverage as the proportion of children having received the primary vaccination before 365 days of age. The acceptable timely coverage of FI was defined as the proportion of children that received all of the 11 vaccinations before 365 days of age. Third, we defined the age-appropriate coverage as the proportion of children having been vaccinated according to the EPI schedule, namely, having received one dose of BCG (between 0 and 1 day of age), three doses of HBV (the first dose starting from 0 and the last one up to 183 days of age), three doses of OPV (the first dose starting from 61 and the last one up to 122 days of age), three doses of DTP (the first dose starting from 92 and the last one up to 153 days of age), and one dose of MR (between 244 and 275 days of age). Given the situation that the due date might be on the weekend or holiday, 7 days later than the due date was defined as acceptable, and was not considered as delay in this study. For the analysis purpose, we considered a month as having 30.5 days in average.

### 2.6. Statistical Analysis

We used STATA 11 (Stata Corp. 2009, Stata statistical software, College Station, TX, USA) for data analysis. First, three kinds of immunization coverage estimates with 95% confidence interval (CI) were calculated by city. Second, we calculated the cumulative probability of being immunized at age for each vaccine dose, through inverse Kaplan–Meier survival function. Besides, we assessed the specific age at which 90% and 95% of children were immunized for each vaccine dose. Children who had not received vaccine doses at 12 months of age were considered as censored. Third, we calculated the proportions of reasons for non-vaccination for each vaccine dose and aggregated them. Fourth, the risk factors for delayed vaccination were identified at the provincial level, using a Cox proportional hazard regression model. The Cox proportional hazard regression model used the demographic or socio-demographic variables as the possible risk factors. The final model was fitted using the backward selection with a cut-off level at *p* < 0.05. The hazard ratios (HR) expressed the rate for a child to be vaccinated at a specific moment in time. HR (if HR > 1) was used to present a higher risk to be vaccinated at a later age compared to the reference group.

### 2.7. Ethical Considerations

This study was approved by the ethical review board of Zhejiang provincial CDC (T-043-R). Written informed consent was obtained from a parent or a legal caregiver of each eligible child enrolled in this study.

## 3. Results

### 3.1. Demographic Characteristics of Surveyed Children

Overall, we surveyed the parents of 2772 eligible children. The proportion of males was 50.3%, and 70.4% of the children were the only child in their household. The proportion of children delivered in hospital was 91.1%. Of all the surveyed mothers, 65.9% were under 30 years of age, 79% had senior middle school background or above, and 86.8% had jobs. The mean of household income per month was 1038.9 US dollars (Table 1).

### 3.2. Vaccination Coverage

The general coverage ranged from 99.4% (95% CI: 99.1–99.7%) for the third dose of DTP (DTP3) and MR to 99.8% (95% CI: 99.6–99.9%) for the first dose of OPV (OPV1) and the first dose of DTP (DTP1). The general FI coverage was 92.6% (95% CI: 91.8–93.3%). The acceptable timely coverage ranged from 96.1% (95% CI: 95.4–96.9%) for MR to 99.6% (95% CI: 99.3–99.8%) for the 1st dose of HBV (HBV1). The acceptable timely FI coverage was 86.8% (95% CI: 85.4–88.3%). The age-appropriate coverage ranged from 25.4% (95% CI: 23.7–27.0%) for BCG to 91.3% (95% CI: 90.3–92.4%) for OPV1 (Table 2).

**Figure 1:**
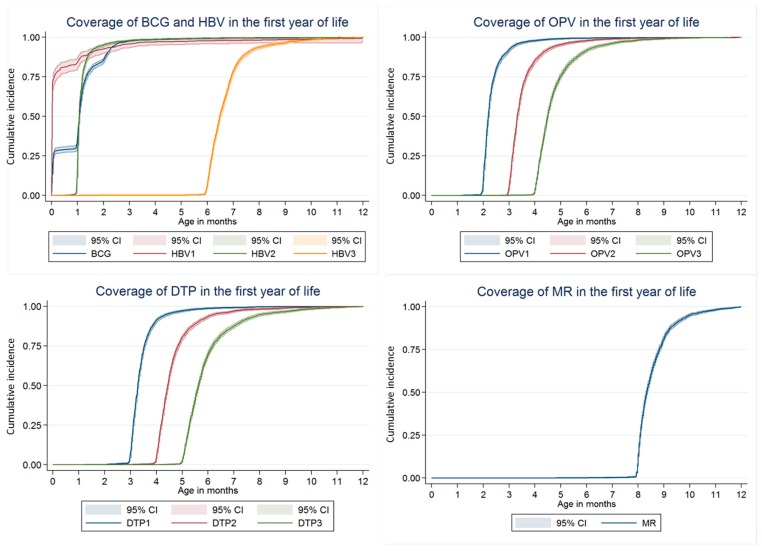
Inverse Kaplan–Meier curves showing the proportion of children immunized with each dose of primary immunizations scheduled in the first year of life among children aged 24–36 months, in Zhejiang province, in 2013 (*N* = 2772).

Ninety percent of the surveyed children had received BCG by 2.3 months of age (95% by 2.6 months). Ninety percent of the surveyed children had received HBV1 by 3.1 months of age (95% by 3.5 months). Ninety percent of the surveyed children had received OPV1 by 2.9 months of age (95% by 3.3 months). Ninety percent of the surveyed children had received DTP1 by 4.0 months of age (95% by 4.6 months). Ninety percent of the surveyed children had received MR by 9.7 months of age (95% by11.0 months) (Figure 1).

### 3.3. Reasons for Non-Vaccination

Table 3 summarized the common reasons reported by parents for 119 non-vaccination doses. The most frequent reason was the parent’s fear of adverse events of immunization (21.8%), followed by the schedule of the immunization clinic being incompatible with working hours (20.2%), and the vaccination contraindications (19.3%).

### 3.4. Risk Factors Related with Delayed Immunization

Table 4 presented the risk factors associated with not being immunized at the appropriate age for the eight different vaccine doses. The results of HBV2, OPV2, and DTP2, were comparable to those of HBV3, OPV3, and DTP3, respectively, and were not presented. Mothers with a lower educational background, or with a job, were associated with delayed immunization. Children delivered at home were more likely to be immunized later. Households with more than one child, or a lower household, income were also found to be risk factors for delayed immunization.

## 4. Discussion

We assessed the coverage of the EPI vaccines scheduled during the first year of life in Zhejiang province. Over 95% of the surveyed children received all primary immunizations before 12 months of age, but few were immunized with these vaccines (except for HBV1 and OPV1) at the recommended age. Several demographic and socio-economic factors related to the timeliness of immunization were identified in this study. These results provided useful information for the EPI improvement in Zhejiang province.

A coverage rate of 90% at a later age may not be sufficient to assure the adequate protection of children, especially for the VPDs with high basic reproduction numbers (such as measles and pertussis), although it has been indicated that rising immunization coverage would increase the timeliness of vaccination simultaneously [17].

Higher rates of untimely vaccinations have been reported in other study settings. For example, Juliet Babirye had conducted a household coverage survey among 821 children aged 10 months to 23 months in Uganda [9]. This study reported that the coverage of receiving all vaccines within the recommended time ranges was 45.6%, and the timely coverage was only 67.5% for measles-containing vaccine. The report from Belgium based on three cross-sectional EPI surveys (2005, 2008, and 2012) indicated that the coverage rates of DTP3 for the three years ranged from 97.9 to 98.7%, but up to 95% of the infants experienced the delayed administrations [18]. Another report from rural Tanzania indicated that the delayed vaccination (>1 month after the recommended age) occurred in 33% of children for BCG, 34% for DTP1, and 69% for DTP3 [19]. Recent studies [20,21] highlighted the necessity of measuring the timeliness of immunization or the up-to-date coverage, as simply considering the coverage at a given age would overestimate the real protection in the relevant population. The implication of untimely immunization is that a pool of children with incomplete or delayed vaccination, are under unnecessary risk of VPDs [9]. With the presence of such a pool of susceptible children, outbreaks can probably occur when the epidemic threshold is exceeded, and it may occur much faster when the delayed immunization is coupled with the low immunization coverage and poor vaccine effectiveness.

Comparing with the results of a similar survey conducted in 2011 for children born between 2008 and 2009 [11], significant improvements on timely vaccination for HBV1 (43.7% vs. 95.1%), OPV1 (45.4% vs. 91.3%), and DTP1 (44.6% vs. 89.7%) were observed. This remarkable evolution may mainly be due to the increased attention for timely vaccination in immunization clinics in Zhejiang province. In fact, vaccinating children at the appropriate age and reducing delays has been prioritized since the 2011 survey. Timely immunization has been considered as an important indicator for assessing the performance of the local immunization program since 2011. Since 2012, the CDCs at province and city levels in Zhejiang province had made active efforts to enhance the timeliness, mainly through raising the awareness of physicians and sending the reminders through cell phones (SMS) at the immunization clinic level which had been indicated effective in other populations or other settings [22,23,24].

There was another explanation for the increased timeliness of vaccination of HBV1. Administration of HBV1 at birth is required for every registered maternity hospital in China. Based on the precondition that the hospital delivery rate has reached almost 100% in Zhejiang province, assuming a mother delivers at a maternity hospital, her child will receive the HBV1 in a timely manner. However, the BCG is recommended to be given at birth and administrated also in maternity hospitals, but it is not mandatory. In fact, a substantial proportion of new babies will receive their BCG in immunization clinic at 1 month of age when they are registered in ZJIIS for the first time. This might be the main reason for the much lower timeliness of BCG. Another explanation for the poor timeliness of BCG is the uncertainty of the efficacy and safety profile of BCG. As we know, BCG can protect tuberculous meningitis and miliary tuberculosis, while its efficacy on preventing the community-acquired tuberculosis is still controversial [25]. On the other hand, the incidence of adverse events on the injection site of BCG vaccination is frequent [26,27,28,29]. Thus, many parents would have hesitations when they decide to vaccinate their children with BCG.

In this study, we found the age-appropriate coverage of DTP1 and MR was lower than the first dose of HBV and OPV. This implied that there was a higher proportion of children with delayed immunization and markedly lower timeliness for consecutive doses of DTP and MR. The delayed MR vaccination was also observed in Switzerland [30], where children spent on average 266 days susceptible to measles from 6 to 24 months of age, and a third of this time was due to the delayed immunization. The threshold of coverage of 95% coverage rate was considered to confer the measles’ herd immunity [31], however, 95% coverage rate was achieved at 11 months of age in this study, which was 2 months later than the scheduled time frame. Furthermore, measles is still a cause of concern in Zhejiang province, which has had measles endemics in the last decade, with the incidence rate over 30/100,000 in 2011. Especially, the proportion of cases aged 8–11 months has increased over time, from 8.5% in 2005, to 23.6% in 2011. As such, we suggest that reaching 95% coverage as early as possible in the children aged ≥ 8 months be a critical step to achieve the goal of measles elimination. As for pertussis, the incidence of pertussis decreased in Zhejiang since 2000, but it still circulates, and the absolute number of reported cases through mandatory notification system is increasing in recent years. It was worth noting that almost a quarter of the cases reported in 2013 were 3–12 months of age. Thus, timely immunization of the pertussis-containing vaccine, such as DTP, is therefore increasingly important.

Despite the high general coverage and increasing timeliness of immunization observed, further steps need to be taken in Zhejiang province to improve adherence to the recommended age of immunization. In this study, we found some risk factors from both the providers and the recipients. First, the schedule of the immunization clinic incompatible with working hours became an important factor of the missing immunization. The WHO recommendation pointed out that the working time of immunization clinics should be extended [32]. The extended time of vaccination sessions may be most beneficial to the children whose mothers have jobs. Second, missing immunizations might be attributed to false contraindications. Although there are few true contraindications to vaccination, vaccination physicians still consider the low-grade fever, cold, diarrhea, vomiting, or other mild illnesses as contraindications for immunization. This inference was demonstrated in previous reports, which also showed the success of the training program in solving these problems [33,34,35]. Another problem of the missing vaccination found in this study was the unnecessary concern about the adverse events. People start and spread rumors about vaccination when adverse events occur. These exaggerations of risk can seriously disrupt immunization programs. We suggest that the endorsement of immunization, and the assurance of vaccine safety, should be sought from the academic institutions, professional associations, politicians, and respected community leaders, through the routine health education program. A previous study demonstrated that educating caregivers could effectively improve the childhood immunization coverage through increasing their knowledge, attitude, and practice towards immunization [36].

This study identified several risk factors related to untimely immunization. First, the low education level can hinder a mother’s communication with physicians, and have a negative influence on childhood immunization through the poor understanding or acceptance of immunization knowledge [37,38]. Second, we assume that mothers with jobs may not have enough time to spare for the childhood immunization, and are less aware of the information on immunization [11]. Third, children delivered at hospitals were more likely to be timely immunized than children delivered at home in this study, which was consistent with the studies done in other settings [39,40]. It is possible that mothers who deliver babies at health facilities may use the health services more frequently, including the childhood immunization. The administration of HBV1 at birth may partly account for the better timeliness of subsequent vaccines. Fourth, we found that a family with more than one child was a negative factor for timeliness of immunization [41]. The main reason is that a family with more children needs to bear a higher cost and much more resources, and it may adversely affect the health service utilization. Moreover, there is very little motivation for parents to prioritize childhood immunization amidst competing demand for time, because the benefits of this activity may not appear immediately [42]. Fifth, children from poorer households were most likely to have the untimely immunizations. This was consistent with the previous report [43], which showed that poverty related factors hindered utilization of vaccination services. It is probably due to the poor accessibility caused by the indirect costs (like transport cost or medication for non-serious adverse events), or deducting wages for work leave for children’s vaccination.

Timeliness of immunization reported in other settings varied substantially. For example, the coverage of delayed immunization ranged from 19% to 78% for the first dose of measles-containing vaccine, and from 18% to 90% for the pertussis-containing vaccine [8,10,18,44]. It might be mainly contributed to the inconsistent definitions applied. Having three definitions of immunization coverage was helpful to understand how general coverage gives only part of the picture when evaluating the EPI. Up to date, a standard definition of the acceptable timing of vaccination has not been developed by the EPI office of China. The definitions used in this study were on the basis of a review of the literature. Besides, the Kaplan–Meier method was useful to visualize the trends of immunization coverage over time, and the Cox proportional hazard regression was useful to explore the risk factors of delayed immunization, adjusting for age at vaccination.

This study was subjected to several limitations. First, we defined non-vaccination as any child without the written evidence of having received specific vaccination from either the immunization card or the vaccination record in ZJIIS. Although this definition could reduce the probability of the recall bias, it also would overestimate the vaccination coverage or timeliness, as those children without the written evidence of vaccination were more likely to be under-immunized or had vaccination delayed. Second, vaccination coverage among children can be influenced by many other factors, including those related to access to the health care, knowledge, attitudes, and practices of parents and providers. However, we could not have controlled for all confounders, due to the unavailability of data. Thus, the influence from those aspects could not be evaluated.

## 5. Conclusions

Common assessment of the EPI is based on the general coverage without considering the timeliness of immunization, which may mask vaccination delays and lead to false assumptions of the protection of VPDs. This study indicated that the timeliness of immunization had been improved since 2011 in Zhejiang province. Nevertheless, necessary steps are still needed to achieve further improvement, especially for MR and DTP, for optimizing the control of relevant VPDs. This study revealed that there was still a substantial proportion of children immunized later than the recommended age, although the general coverage was optimal. There are some possible interventions to improve the timeliness of immunization. First, the timeliness of immunization should be considered as another important indicator of EPI performance assessment. Second, the Reaching Every District (RED) [45] strategy (like outreach vaccination services or remind/recall services) may be effective in improving the coverage and timeliness among the children who still remain hard to reach. Third, the importance of adhering to the recommended schedule needs to be emphasized to parents, through the health education program.

## Figures and Tables

**Table 1:** Summary distribution of the demographic and socio-economic characteristics of children aged 24–36 months, in Zhejiang province.

Variables	No. of Children (%) *N = 2772*
Sex of child	
Male	1395 (50.3)
Female	1377 (49.7)
Number of children in the surveyed household	
1	1952 (70.4)
2	651 (23.5)
≥3	169 (6.1)
Place of delivery	
Hospital	2525 (91.1)
Home	247 (8.9)
Age of mother (years)	
<30	1826 (65.9)
≥30	946 (34.1)
Maternal education level	
<senior middle school ^a^	583 (21.0)
≥senior middle school	2189 (79.0)
Maternal employment status	
Home fulltime	367 (13.2)
Employed	2405 (86.8)
Residence	
Urban	1362 (49.1)
Rural	1410 (50.9)
Immigration status	
Resident	1661 (59.9)
Migrant	1111 (40.1)
Family income per month (US dollars) ^b^	1038.9 ± 15.8

^a^ Senior middle school starts from grade 10 to grade 12; ^b^ Continuous variable, mean ± SD.

**Table 2:** Immunization coverage in general, according to acceptable timing, at the appropriate age among children aged 24–36 months, in Zhejiang province (*N* = 2772).

Vaccine Dose	General Coverage		Acceptable Timely Coverage		Age-Appropriate Coverage	
	*n*	% (95% CI)	*n*	% (95% CI)	*n*	% (95% CI)
BCG	2759	99.5 (99.3–99.8)	2746	99.1 (98.7–99.4)	703	25.4 (23.7–27.0)
HBV1	2763	99.7 (99.5–99.9)	2760	99.6 (99.3–99.8)	2635	95.1 (94.3–95.9)
HBV2	2762	99.6 (99.4–99.9)	2755	99.4 (99.1–99.7)	2619	94.5 (93.7–95.3)
HBV3	2760	99.6 (99.3–99.8)	2751	99.2 (98.9–99.6)	2118	76.4 (74.8–77.9)
OPV1	2765	99.8 (99.6–99.9)	2754	99.4 (99.1–99.7)	2534	91.3 (90.3–92.4)
OPV2	2764	99.7 (99.5–99.9)	2746	99.1 (98.7–99.4)	2327	83.9 (82.5–85.4)
OPV3	2762	99.6 (99.4–99.9)	2732	98.6 (98.1–99.0)	2062	74.4 (72.7–76.0)
DTP1	2765	99.8 (99.6–99.9)	2746	99.1 (98.7–99.4)	2487	89.7 (88.6–90.9)
DTP2	2762	99.6 (99.4–99.9)	2732	98.6 (98.1–99.0)	2182	78.7 (77.2–80.3)
DTP3	2755	99.4 (99.1–99.7)	2701	97.4 (96.9–98.0)	1865	67.3 (65.5–69.0)
MR	2756	99.4 (99.1–99.7)	2665	96.1 (95.4–96.9)	2172	78.4 (76.8–79.9)
FI ^a^	2566	92.6 (91.8–93.3)	2406	86.8 (85.4–88.3)	-	-

^a^ Full immunization.

**Table 3:** Reasons for non-vaccination among children aged 24–36 months, in Zhejiang province.

Reasons	BCG (*n* = 12)		HBV1 (*n* = 9)		HBV2 (*n* = 10)		HBV3 (*n* = 12)		OPV1 (*n* = 7)		OPV2 (*n* = 8)		OPV3 (*n* = 10)		DTP1 (*n* = 7)		DTP2 (*n* = 10)		DTP3 (*n* = 17)		MR (*n* = 16)		All (*n* = 119)	
	*n*	%	*n*	%	*n*	%	*n*	%	*n*	%	*n*	%	*n*	%	*n*	%	*n*	%	*n*	%	*n*	%	*n*	%
Was not aware that the child needed this vaccination	1	7.7					3	25.0	1	14.3			1	10.0	2	28.6	1	10.0	3	17.6			12	10.1
The immunization clinic was overcrowded					2	20.0	2	16.7	1	14.3	1	12.5	1	10.0	1	14.3	1	10.0	2	11.8	1	6.25	12	10.1
The schedule of the immunization clinic was incompatible with working hours					6	60.0	4	33.3	1	14.3	2	25	2	20.0			2	20.0	3	17.6	4	25	24	20.2
Fear of adverse events	9	69.2							2	28.6	1	12.5	3	30.0	3	42.9	3	30.0	3	17.6	2	12.5	26	21.8
The child was sick when the vaccine was due					1	10.0	2	16.7			2	25			1	14.3	1	10.0	3	17.6	7	43.8	17	14.3
The physicians had contraindicated the vaccine			9	100.0	1	10.0	1	8.3	2	28.6	2	25	2	20.0			2	20.0	2	11.8	2	12.5	23	19.3
Considered vaccination not important	3	23.1											1	10.0					1	5.88			5	4.2

**Table 4:** Hazard ratio (with 95% CI) for untimely immunization of eight different vaccine doses among children aged 24–36 months, in Zhejiang province (Cox proportional hazard regression, final models, *N* = 2772).

Demographic and Socio-Economic Variables		BCG	HBV1	HBV3	OPV1	OPV3	DTP1	DTP3	MR
Mother’s education level	<senior middle school	Ref	Ref	Ref	Ref	Ref	Ref	Ref	Ref
	≥senior middle school	NS	0.91 (0.87–0.99) *	0.85 (0.80–0.92) *	NS	NS	NS	0.84 (0.75–0.92) *	0.79 (0.72–0.87) **
Mother’s occupation	Home fulltime	Ref	Ref	Ref	Ref	Ref	Ref	Ref	Ref
	Employed	NS	NS	1.57 (1.38–1.70) **	NS	1.25 (1.17–1.40) *	1.05 (1.00–1.09) *	1.42 (1.35–1.55) **	1.61 (1.40–1.74) **
Number of children in the surveyed household	1	Ref	Ref	Ref	Ref	Ref	Ref	Ref	Ref
	2	1.06 (0.86–1.14)	NS	1.05 (0.91–1.17)	NS	1.07 (0.92–1.25)	NS	NS	NS
	≥3	1.18 (1.12–1.31) *	NS	1.12 (1.03–1.19) *	NS	1.09 (1.03–1.15) *	NS	NS	NS
Place of delivery	Hospital	Ref	Ref	Ref	Ref	Ref	Ref	Ref	Ref
	Home	NS	1.76 (1.43–2.21) **	NS	NS	NS	1.17 (1.10–1.27) *	NS	1.38 (1.20–1.51) **
Family income per month ^a^		NS	NS	0.79 (0.72–0.90) **	NS	0.88 (0.82–0.96) *	NS	0.85 (0.80–0.93) *	0.75 (0.69–0.88) **

^a^ Continuous variable, increasing direction; HR (hazard ratio) presented in bold were significant with * *p* < 0.05; ** *p* < 0.01, respectively; Ref: reference; NS: no significant.
